# McKittrick–Wheelock Syndrome: A Rare Case of Secretory Diarrhea

**DOI:** 10.1155/2022/2097364

**Published:** 2022-12-05

**Authors:** Mohammad Nabil Rayad, Noreen Mirza, Maria Bernardeth Herrera-Gonzalez, Yatinder Bains, Sarahi Herrera-Gonzalez

**Affiliations:** ^1^Department of Internal Medicine, St Michael's Medical Center, 111 Central Ave, Newark, NJ 07102, USA; ^2^Division of Health Sciences, University of Monterrey, Avenida Ignacio Morones Prieto 4500-Pte, San Pedro Garza Garcia, Nuevo Leon 66238, Mexico; ^3^Department of Gastroenterology, St Michael's Medical Center, 111 Central Ave, Newark, NJ 07102, USA

## Abstract

McKittrick–Wheelock syndrome commonly presents with a triad of chronic secretory diarrhea, electrolyte disturbances, and renal failure. Secretory diarrhea is due to active ion secretion secondary to secretagogue secretion (cyclic adenosine monophosphate and prostaglandin E2). The mainstay of treatment for these lesions is surgical since it will arrest the loss of electrolytes that may lead to serious clinical consequences. Nonsteroidal anti-inflammatory drugs (NSAIDs) such as indomethacin may be used to decrease electrolyte secretion in patients that desire a nonsurgical approach. Our patient is unique in that this is the first case of a tubular adenoma with high-grade dysplasia leading to MWS and progressing to circulatory collapse with severe electrolyte disturbances. Aggressive replacement of fluids and electrolytes is essential to the survival of these patients.

## 1. Introduction

Diarrhea is multifactorial in nature and is defined by an increase in stool frequency, volume, or fluidity. Diarrhea is caused by an imbalance in two processes which occur simultaneously including either an increase in the level of section or a decrease in the amount of absorption [1]. There are two main types of diarrheas including secretory or osmotic. Osmotic diarrhea occurs due to production of a gradient by certain nonabsorbable substances [1]. Decreased water absorption and increased secretion due to disrupted epithelial transport of electrolytes cause secretory diarrhea [1]. In secretory diarrhea, stool volume persists regardless of the fasting state and the osmolar gap is usually <50; nonetheless, an osmolar gap of <100 is highly suggestive of secretory diarrhea [2].

McKittrick–Wheelock syndrome is a rare cause of secretory diarrhea and was first reported in 1954 by McKittrick and Wheelock [3]. It is caused by a secretory colorectal tumor that is most frequently a villous adenoma and less commonly adenocarcinoma. These tumors tend to affect various age groups and have a high frequency of malignant conversion [3]. The most common location for these tumors includes the rectum. Furthermore, the complications of these secretory colorectal tumors include severe fluid and electrolyte losses [3]. The mainstay of treatment for these pathological entities includes surgical resection [3]. We present a case of a tubular adenoma with high-grade dysplasia as a cause of secretory diarrhea with hypovolemic shock.

## 2. Case Presentation

A 74-year-old male with history of hypertension, iron deficiency anemia, schizophrenia, and seizure disorder was brought to the hospital from a nursing home for the evaluation of watery diarrhea for several days followed by hypotension. As per reports from the nursing home, the patient had dementia at baseline oriented only to self and in recent months had become bedridden. On arrival to the hospital, the patient was presumed to be in septic shock with hypotension, tachycardia, and hypothermia. On physical examination, the patient was confused and severely malnourished, with mild diffuse abdominal tenderness and bilateral sacral and buttock pressure wounds. Initial evaluation revealed gross abnormalities with WBC of 106 10^*∗*^3/*μ*L(4.4–11.00 10^*∗*^3/*μ*L), Hb of 9.4 g/dL (13.5–17.5 g/dL), MCV of 78 fL (81.2–95.1 fL), platelets of 889 10^*∗*^3/*μ*L(150–450 10^*∗*^3/*μ*L), BUN of 59 mg/dL (6–24 mg/dL), Cr of 3.2 mg/dL (0.6–1.2 mg/dL), Na of 138 mmol/L (136–145 mmol/L), K of 4.8 mmol/L (3.5–5.3 mmol/L), Cl of 103 mmol/L (98–110 mmol/L), HCO3 of 12 mmol/L (20–31 mmol/L), and LA of 11.4 mmol/L (0–2 mmol/L). He received resuscitation with intravenous fluids and broad-spectrum antibiotics to which he did not successfully respond and required initiation of intravenous vasopressors. Septic workup was relevant for urinary tract infection. During the first days of hospitalization, a rectal tube was placed due to high volume diarrhea where daily output ranged from 3.2–4.5 liters daily.

Stool studies resulted negative for *Clostridium difficile* on three different occasions, cultures for Salmonella, Shigella, Campylobacter, E. coli O157:H7 were negative, as well as ova, parasite and cryptosporidium, Giardia lamblia antigens. Stool sodium was 89 mEq/L, and K was 21 mEq/L with a stool osmolar gap of 70 mOsm/kg. Fecal lactoferrin was 41.69 (*μ*g/mL).

**Figure 1:**
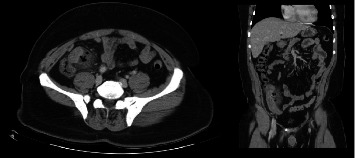
Computed tomography of the abdomen demonstrating mural thickening on the descending and ascending colon with adjacent fatty stranding.

Computed tomography revealed mural thickening on the descending and ascending colon with adjacent fatty stranding (Figure 1).

**Figure 2:**
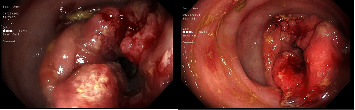
Flexible sigmoidoscopy showing a fungating partially obstructing large mass in the distal sigmoid colon, 25 cm away from anal verge.

Extensive workup for secretory diarrhea was obtained includinggastrin 66 pg/mL (0–115 pg/mL), urine 5HIAA 0.6 mg/24 hrs (0.0–14.9 mg/24 hrs), serotonin <5 ng/mL (21–321 ng/mL), AM cortisol 17.2 *μ*g/dl (7–25 *μ*g/dl), ACTH pg/mL (7.2–63.3 pg/mL), Insulin <1.0 *μ*U/mL (0–17 *μ*U/mL), C-Peptide 3.2 ng/mL (1.1–4.4 ng/mL), glucagon levels were not available at our laboratory. Despite aggressive fluid resuscitation, the patient remained hypotensive on vasopressors. A fasting challenge was done for 48 hrs with no change in diarrhea volume. Octreotide was initiated with three times daily subcutaneous octreotide injections and up-titrated up to 300 mcg without changes on output and hemodynamics. Flexible sigmoidoscopy revealed a fungating, partially obstructing large mass in the distal sigmoid colon about 25 cm from the anal verge (Figure 2).

**Figure 3:**
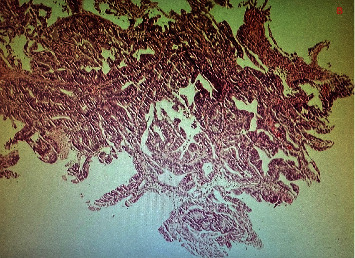
H&E slide: fragments of a tubular adenoma with high-grade dysplasia (intramucosal adenocarcinoma).

Biopsies demonstrated fragments of a tubular adenoma with high-grade dysplasia (intramucosal adenocarcinoma) (Figure 3).

Oncology and surgical consultations were requested; however, the patient was not deemed a surgical or chemotherapy candidate due to functional status and poor overall condition. After discussion with family members, it was decided to withdraw life support and the patient succumbed to his disease.

## 3. Discussion

Diarrhea is multifactorial in nature and is defined by an increase in stool frequency, volume, or fluidity [1]. It is primarily caused due to the inability of the intestinal lumen to absorb water [1]. However, there are two main processes that can contribute to the development of diarrhea, the first being secretion and the second being absorption. These two mechanisms occur simultaneously; however, absorption tends to take longer. A decrease in the level of absorption or an increase in the level of secretion will lead to the accumulation of water in the lumen and therefore diarrhea will occur [1].

Diarrhea can be further categorized as osmotic or secretory. Osmotic diarrhea occurs when unabsorbed substances draw water from the plasma into the bowel lumen causing the production of an osmotic gradient [1]. On the other hand, decreased water absorption and increased secretion due to disrupted epithelial transport of electrolytes cause secretory diarrhea [1]. To distinguish the two main categories of diarrhea described above, it is imperative to calculate the fecal osmotic gap [1]. The fecal osmotic gap is calculated by the difference of the lumen osmolality, which is approximately equal to the body fluid osmolality (∼290 mOsm/kg) and the osmolality of fecal electrolytes (contributed by twice the sum of potassium and sodium) in the lumen [2]. In osmotic diarrhea, the presence of unabsorbed substances (solutes or unmeasured cations) attract water and thus dilute fecal sodium and potassium concentrations. The stool osmolar gap in osmotic diarrhea is therefore >125 mOsm/kg [4]. In secretory diarrhea, the colon is unable to adjust for stool electrolytes, and thus the stool osmolar gap is typically defined as <50 mOsm/kg or <100 mOsm/kg, which varies according to the literature reviewed [4, 5]. The response to the fasting state is also able to differentiate osmotic versus secretory diarrhea. When considering osmotic diarrhea in a fasting state, stool volume decreases, whereas in secretory diarrhea, stool volume persists regardless of the fasting state [1]. The total daily volume also differs between the two types of diarrhea with secretory having large volume watery stools of more than 1 liter per day versus osmotic diarrhea having less than 1 liter per day [6]. In our case, the patient's stool osmotic gap was 70 mOsm/kg, without improvement in the fasting state and with a total volume of over 3000 ml/day, supporting the diagnosis of secretory diarrhea.

Secretory diarrhea is typically caused by endocrine disturbances including carcinoid tumors, gastrinomas, VIPomas, and medullary carcinomas of the thyroid [4]. Other common causes include diabetes-related diarrhea, alcohol, and factitious diarrhea (commonly from bisacodyl laxative abuse) [4]. Infections, bile acid malabsorption, inflammatory bowel diseases (Crohn's disease, microscopic colitis, and ulcerative colitis), disordered regulation (postvagotomy), neoplastic lesions (typically lymphoma, villous adenoma, and colon carcinoma), and idiopathic causes can also give origin to secretory diarrhea [1].

While most colonic polyps are asymptomatic, severe secretory diarrhea has been reported in the past caused due to the presence of colonic adenomas [7, 8]. In general, tubular colon adenomas are known to be precancerous and could undergo malignant transformation. This contrasts with hyperplastic polyps that have no malignant potential. Villous features in a polyp include long “finger-like” protrusions [9]. Tubular adenomas are known to have less than 25% villous features. When a polyp has >75% villous features, it is classified as a villous adenoma. Tubulovillous adenomas are, as the name suggests, adenomas that carry both tubular and villous features [8]. Malignancy risk tends to increase linearly with an increase in the size of the polyp, positive family history of colon cancer, histological type, and degree of dysplasia [10, 11].

McKittrick–Wheelock syndrome is a rare cause of secretory diarrhea and was first reported in 1954 by McKittrick and Wheelock [3]. It is caused by a secretory colorectal tumor that is most frequently a villous adenoma and less commonly adenocarcinoma [11, 12]. Severe secretory diarrhea along with electrolyte disturbances in the form of McKittrick–Wheelock syndrome has also been reported in villous adenomas [8]. Villous adenomas that are ranging from 4 to 18 cm are considered large, and they can cause secretory diarrhea of 500 to 3000 mL/24 hours [4]. The mechanism of action that has been proposed for adenomatous polyps causing large volume secretory diarrhea is due to active ion secretion secondary to secretagogue secretion by adenomas/adenocarcinomas [13]. The secretagogues that are responsible include cyclic adenosine monophosphate and prostaglandin E2 [13]. These lead to the flux of electrolytes and fluids into the lumen [13]. McKittrick–Wheelock syndrome classically presents with a triad consisting of chronic diarrhea, electrolyte disturbances (most commonly hypokalemia), and renal failure [12]. An increase in the number of goblet cells and prostaglandin E2 is responsible for this type of diarrhea [4]. The large surface area of the larger tumors essentially leads to an increase in fluid secretion that triumphs the normal mucosa's ability to reabsorb the presence of extra fluid [14]. Furthermore, the presence of COX-2 expression is known to be higher in tubulovillous adenoma [14].

To work up a patient that presents with chronic diarrhea, it is imperative to conduct a thorough history to identify certain drugs and dietary factors that may be contributing to the patient presentation [13]. Metabolic disorders are routinely diagnosed via blood tests. Infectious causes can typically be ruled out by the examination of stool studies [14]. The most essential step in diagnosis for a patient with chronic diarrhea is colonoscopy to search for the presence of signs of inflammatory bowel disease or the presence of a polyp and colonic neoplasms [14].

The mainstay of treatment for these lesions is surgical. Typical surgical options include transanal resection using an approach, that is, presacral mucosectomy, and abdominal surgery for large tumors [8]. This is the ultimate treatment that will arrest the loss of electrolytes that may lead to serious clinical consequences. In patients that have McKittrick–Wheelock syndrome, nonsteroidal anti-inflammatory drugs such as indomethacin may be used to decrease the electrolyte secretion in patients that desire a conservative nonsurgical approach [8].

## 4. Conclusion

McKittrick–Wheelock syndrome is a rare syndrome and is most commonly caused by villous adenomas. Our patient is unique in that this is the first ever reported case of a tubular adenoma with high-grade dysplasia leading to a McKittrick–Wheelock presentation and importantly progressing towards circulatory collapse with severe electrolyte disturbances. Aggressive replacement of fluids and electrolytes is essential to the survival of these patients, and it is imperative that clinicians be aware of this rare diagnosis.
